# Cross-neutralisation of viruses of the tick-borne encephalitis complex following tick-borne encephalitis vaccination and/or infection

**DOI:** 10.1038/s41541-017-0009-5

**Published:** 2017-03-13

**Authors:** Alexander J. McAuley, Bevan Sawatsky, Thomas Ksiazek, Maricela Torres, Miša Korva, Stanka Lotrič-Furlan, Tatjana Avšič-Županc, Veronika von Messling, Michael R. Holbrook, Alexander N. Freiberg, David W. C. Beasley, Dennis A. Bente

**Affiliations:** 1 Department of Microbiology and Immunology, University of Texas Medical Branch, 301 University Boulevard, Galveston, TX 77555-0610 USA; 2 Institute for Human Infections and Immunity, University of Texas Medical Branch, 301 University Boulevard, Galveston, TX 77555-0609 USA; 3 Department of Veterinary Medicine, Paul-Ehrlich-Institut, Paul-Ehrlich-Straße 51-59, 63225 Langen, Hessen, Germany; 4 Department of Pathology, University of Texas Medical Branch, 301 University Boulevard, Galveston, TX 77555-0609 USA; 5 Institute of Microbiology and Immunology, Faculty of Medicine, University of Ljubljana, Zaloška 4, Ljubljana , 1000 Slovenia; 6 Department of Infectious Diseases, University Medical Center Ljubljana, Japljeva 2, Ljubljana , 1525 Slovenia; 7 Integrated Research Facility, National Institutes of Health, 8200 Research Plaza, Frederick, MD 21702 USA; 8 Sealy Center for Vaccine Development University of Texas Medical Branch, 301 University Boulevard, Galveston, TX 77555-0609 USA; 9 Present Address: CSIRO Australian Animal Health Laboratory, 5 Portarlington Road, Geelong, VIC 3220 Australia

## Abstract

The tick-borne encephalitis complex contains a number of flaviviruses that share close genetic homology, and are responsible for significant human morbidity and mortality with widespread geographical range. Although many members of this complex have been recognised for decades, licenced human vaccines with broad availability are only available for tick-borne encephalitis virus. While tick-borne encephalitis virus vaccines have been demonstrated to induce significant protective immunity, as determined by virus-neutralisation titres, vaccine breakthrough (clinical infection following complete vaccination), has been described. The aim of this study was to confirm the cross-neutralisation of tick-borne flaviviruses using mouse immune ascitic fluids, and to determine the magnitude of cross-neutralising antibody titres in sera from donors following tick-borne encephalitis vaccination, infection, and vaccine breakthrough. The results demonstrate that there is significant cross-neutralisation of representative members of the tick-borne encephalitis complex following vaccination and/or infection, and that the magnitude of immune responses varies based upon the exposure type. Donor sera successfully neutralised most of the viruses tested, with 85% of vaccinees neutralising Kyasanur forest disease virus and 73% of vaccinees neutralising Alkhumra virus. By contrast, only 63% of vaccinees neutralised Powassan virus, with none of these neutralisation titres exceeding 1:60. Taken together, the data suggest that tick-borne encephalitis virus vaccination may protect against most of the members of the tick-borne encephalitis complex including Kyasanur forest disease virus and Alkhumra virus, but that the neutralisation of Powassan virus following tick-borne encephalitis vaccination is minimal.

## Tick-borne encephalitis: One vaccine protects against multiple flaviviruses

Prior exposure to tick-borne encephalitis virus confers immunity to distinct, but genetically related, significant human pathogens. The tick-borne encephalitis complex comprises multiple ‘flaviviruses’; however, a licensed vaccine only exists for one: tick-borne encephalitis virus (TBEV). Alexander McAuley, of the University of Texas Medical Branch, USA, and collaborators tested human serum samples from donors who had either received the TBEV vaccine, been infected with the virus naturally, or had contracted the virus despite prior vaccination. The team tested the sera against six genetically related flaviviruses, looking for antibodies that indicate viral immunity. The results showed that TBEV exposure induce production of antibodies that inhibit other flaviviruses, the efficacy of which decreases as the genetic variation from TBEV increases. Additionally, sera from infected individuals generated more antibodies than sera from vaccinated donors. This study provides an insight into the relationship between genetic similarity and vaccine cross-protection.

## Introduction

The tick-borne encephalitis (TBE) complex is a group of enveloped, non-segmented, positive-sensed single-stranded RNA viruses within the genus *Flavivirus* (family *Flaviviridae*).^1^ Many of the viruses of the TBE complex are significant human pathogens, such as tick-borne encephalitis virus (TBEV), Omsk haemorrhagic fever virus (OHFV), Kyasanur forest disease virus (KFDV), and Powassan virus (POWV), and have a wide global distribution spanning North America, Europe, the Middle East, and Asia.^2^ The viruses display substantial sequence similarity at both the nucleotide and amino-acid level, with the two most genetically distant members of the complex (POWV and Alkhumra haemorrhagic fever virus [AHFV]) sharing 67.3% nucleotide identity between their genomes, and 75.6% amino-acid identity between their polyproteins.^3^ Despite the high degree of sequence similarity, members of the TBE complex cause a spectrum of diseases, ranging from encephalitis (e.g., TBEV, louping ill virus (LIV), POWV) to haemorrhagic fever (e.g., OHFV, KFDV, AHFV).^1^ Outbreaks of many of the tick-borne flaviviruses are quite common, with TBEV causing an average of 2,600 cases annually in Europe, KFDV causing an average of 400–500 cases per year in India, and AHFV causing approximately 20 cases a year in the Middle East.^4–6^ The high mortality associated with a number of the members of the TBE complex has led to their classification as risk-group four pathogens in non-endemic countries, requiring work with these viruses to be restricted to Biosafety Level 4 (BSL-4) facilities.^7^

Early, comprehensive cross-reactivity and cross-neutralisation studies were performed by Clarke, Gresíkova & Sekeyová, Stephenson *et al.* and Calisher *et al.* showing clear relationships of differing degrees between the viruses, and allowing for the delineation of distinct serocomplexes.^8–14^ Cross-protection studies by Casals and others demonstrated that the serological relationships were not just academic, but that to varying degrees cross-reactivity and cross-neutralisation could lead to cross-protection *in vivo*.^15, 16^ Genetic characterisation of members of the TBEV serocomplex has demonstrated that antigenic determination of relationships between members was largely accurate.^1^ In addition, genetic analysis was able to more carefully define the three subtypes of TBEV, namely the Far-Eastern, Siberian and European subtypes.^17^

Owing to the dearth of specific treatments for flaviviruses in general, vaccination remains the key method of medical intervention. A number of vaccines against members of the TBE complex have been developed, but only a few have been licenced for widespread use. Since its discovery, TBEV has been a major target for vaccine development, and remains the only tick-borne flavivirus for which a vaccine is widely available.^1, 18^ Early vaccines were based upon formalin-inactivated emulsified mouse brain, and were demonstrably immunogenic, but were gradually phased out due to concerns about adverse reactions towards the mouse brain components.^19–21^ By the late 1950s, alternatives to mouse brain-derived vaccines were being developed, including those derived from embryonated eggs and tissue culture.^22–24^ For a while, a live, attenuated vaccine against multiple tick-borne flaviviruses appeared possible using the attenuated Langat virus (LGTV) TP21 strain, however, in spite of a few trials in human volunteers, the vaccines did not proceed to licensure due to the development of neurological complications in a number of vaccinated individuals.^25, 26^ Today there are four approved vaccines for TBEV: two European vaccines, FSME-Immun/TicoVax (Pfizer Pharma, Berlin, Germany) and Encepur (Chiron Behring, Marburg, Germany); and two Russian vaccines, TBE Moscow (Chumakov Institute, Moscow, Russia) and Encevir (Microgen, Tomsk, Russia).^27^ The current vaccines are derived from primary chicken embryo cells infected with either TBEV Neudörfl (European subtype; FSME-Immun), TBEV K23 (European subtype; Encepur), TBEV Sofjin (Far-Eastern subtype; TBE Moscow), or TBEV Strain 205 (Far-Eastern subtype; Encevir), are formalin-inactivated prior to filtration and purification, and are adjuvanted using aluminium hydroxide.^27^ Each of these strains share over 80% nucleotide and 93% amino-acid identity across their genomes and polyproteins, respectively.

Although the TBEV vaccines have been shown to be relatively efficacious in the field, vaccine breakthroughs, in which patients develop clinical TBEV infection despite vaccination, have been described.^28, 29^ These vaccine breakthrough events tend to be in older individuals (>60 years), and are associated with an anamnestic immune response, suggesting that vaccine-induced immune responses, while present, were not sufficiently protective to prevent infection.^29–31^

Vaccines against other TBE complex viruses have been generated using similar techniques: A mouse brain-derived vaccine against OHFV was produced in Russia during the 1950s, but little information is available about its development or efficacy.^32^ Similarly, vaccines against KFDV were generated from a number of substrates, including formalin-inactivated emulsified mouse brain, embryonated eggs, and tissue culture.^33–35^ Studies with the tissue culture-derived vaccine in 87 human volunteers showed variable immunogenicity, with only 50% of vaccinees demonstrating neutralising antibodies, with a further 22.5% showing partial protection.^36^ In a larger-scale investigation with 214 volunteers, a seroconversion rate of only 59% was generated, with reduced vaccine efficacy seemingly arising from pre-existing antibodies to other flaviviruses (e.g., dengue virus, West Nile virus and Japanese encephalitis virus).^37, 38^ Similar approaches were taken for the development of a Louping ill vaccine for use in sheep, with the first vaccine derived from formalin-inactivated sheep brain homogenate, and the more recent vaccine produced in tissue culture.^39, 40^

Studies investigating cross-protection following vaccination with commercial vaccines against TBE complex viruses have been somewhat limited, but have increased over the last few years. Soon after the identification of KFDV in the late 1950s, epidemiological studies were carried out to determine whether the then-recently developed, formalin-inactivated mouse brain-derived TBEV vaccine would generate protective immune responses against KFDV infection.^41^ In these studies, protection of human vaccinees against KFDV infection was minimal, and the use of the TBEV vaccine against KFDV infection was abandoned in favour of the previously described KFDV-derived vaccines. More recently, a number of studies have looked at the current TBEV vaccines with respect to their ability to generate effective, cross-protective immune responses towards a spectrum of TBEV strains and OHFV.^42, 43^ These studies have shown that the immune responses generated following vaccination are protective against OHFV and the different subtypes of TBEV.

To date there have been no comprehensive studies to determine the extent of cross-neutralisation of tick-borne flaviviruses following TBEV vaccination. This is particularly important for viruses such as KFDV and AHFV, viruses that cause severe disease in humans, but for which licenced vaccines are not readily available, or have very limited distribution. In addition, although comparisons have been made between antibody responses generated following TBEV vaccination and infection, as well as after vaccine breakthrough, the effect these antibody profiles have on cross-neutralisation of TBE complex flaviviruses have yet to be fully elucidated.^44–47^

## Results

### Cross-neutralisation with hyperimmune mouse ascitic fluids

**Fig. 1:**
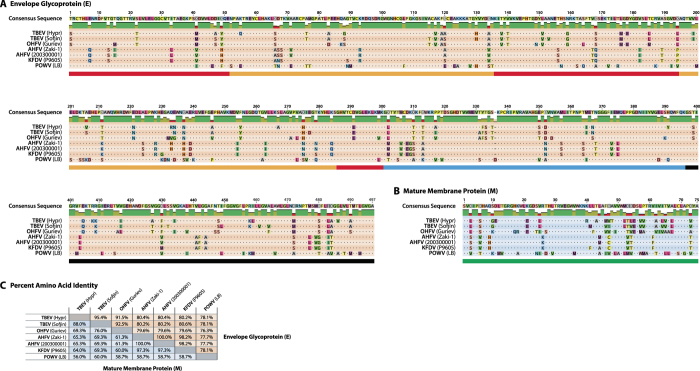
Alignment and percent amino-acid identity between tick-borne flavivirus membrane (M) and envelope (E) proteins. Amino-acid sequences for the envelope glycoprotein (E; Fig. 1a) and mature membrane protein (M; Fig. 1b) of seven tick-borne flavivirus strains (listed in Table 1) were aligned using MUSCLE in Geneious R9 (Biomatters, Auckland, New Zealand). Domains of E (EI, EII, EIII and stem-helix) are denoted by *red*, *orange*, *blue* and *black bars*, respectively, underneath the alignment. The mature M protein sequence is underlined with a *green bar*. The percent amino-acid identity between the different virus E and M proteins is given in Fig. 1c

Previous studies have used hyperimmune mouse ascitic fluids to compare the antigenicity of tick-borne flaviviruses with a view to grouping serologically-related viruses together.^8–13, 15^ We first wanted to confirm these previous studies with our own mouse immune ascitic fluids (MIAFs) from the World Reference Collection of Emerging Viruses and Arboviruses (WRCEVA) at University of Texas Medical Branch (UTMB), and to evaluate the relationship between neutralisation and amino-acid sequence variation within M and E, the two structural proteins present on the surface of the virion, and thus the viral factors involved in antibody-mediated virus neutralisation. Figures 1a, b show the E and M amino-acid alignments, respectively, for the viruses included in this study, with percent amino-acid identities for each protein (Fig. 1c).

**Fig. 2:**
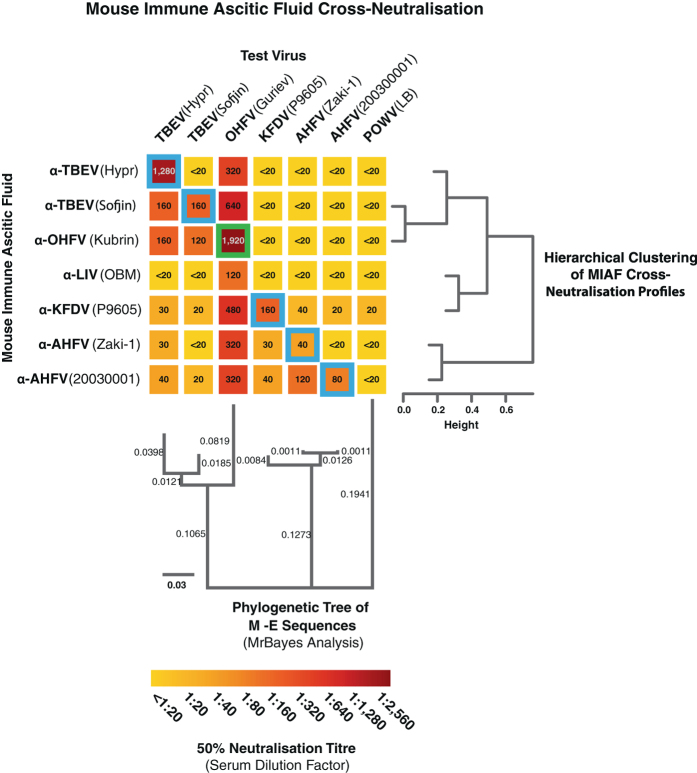
Cross-neutralisation of tick-borne flaviviruses with virus-specific mouse immune ascitic fluids (MIAFs). Seven tick-borne flaviviruses (listed in Table 1) were neutralised by MIAFs raised against homologous and heterologous virus strains, with the neutralisation (NT_50_) titres presented in a heatmap. Homologous neutralisation is highlighted by *blue boxes* around the neutralisation titres (the *green box* for OHFV indicates a heterologous strain). The donor viruses were grouped based upon MrBayes phylogenetic analysis of the M-E amino-acid sequences (analysis performed using Geneious R9), while the MIAF cross-neutralisation responses were grouped based upon hierarchical clustering (performed using Mathematica v10; Wolfram Research, Champaign, IL)

Neutralisation of the viruses listed in Table 1 with MIAFs raised against TBEV Hypr, TBEV Sofjin, OHFV Kubrin, LIV OBM, KFDV P9605, AHFV Zaki-1 and AHFV 200300001 showed similar cross-neutralisation profiles to previously published data, with the addition of both AHFV MIAF and virus (Fig. 2). An anti-LIV MIAF was included as the virus clusters with the TBEV strains at the M-E sequence level, but is primarily a virus of sheep, and has only occasionally caused disease in humans.^48^ Significant variability was observed with homologous samples, suggesting that the MIAF antibody levels differed substantially. Hierarchical clustering of the neutralisation profiles for each MIAF revealed three main clusters: the first consisting of the anti-TBEV Hypr, anti-TBEV Sofjin and anti-OHFV Kubrin MIAFs; the second containing the anti-LIV OBM and anti-KFDV P9605 MIAFs; and the third containing the two anti-AHFV MIAFs (Zaki-1 and 200300001). This clustering is similar to that observed for the MrBayes analysis of the M-E sequence for each of the viruses, leading to a similar order of viruses and MIAFs in the heatmap.

**Table 1:** Viruses used for cross-neutralisation studies and MIAF production

Virus	Strain	Accession number	Used w/MIAF	Used w/human sera	MIAF reference number
TBEV	Hypr	U39292	Yes	Yes	R181
TBEV	Sofjin	AB062064	Yes	Yes	R184
OHFV	Guriev	AB507800	Yes	Yes	—
OHFV	Kubrin	AY438626	—	—	R158
AHFV	Zaki-1	JF416956	Yes	Yes	R204
AHFV	200300001	JF416954	Yes	No	R050
KFDV	P9605	HM055369	Yes	Yes	R157
POWV	LB	L06436	Yes	Yes	POWV LB MIAF

### Cross-neutralisation with human vaccinee, infectee and vaccine breakthrough Sera

In order to further characterise cross-neutralisation potential of tick-borne flaviviruses following exposure to TBEV antigens, human serum samples were obtained from donors who had either received a full course of licenced TBEV vaccine, had been naturally infected with TBE virus, or had been infected in spite of prior vaccination (vaccine breakthrough; Table 2). For some of the donors, multiple samples were available from different timepoints following vaccination or infection. In total, there were 19 donors who had received vaccine only (20 samples), 13 donors who had been naturally infected with TBEV (18 samples), and 5 vaccine breakthrough donors (11 samples).

**Table 2:** Donor information for TBEV human serum samples

	Vaccinee samples	Infectee samples	Vaccine breakthrough samples
Sex	5M, 10F, 4Unk (67% female)	8M, 5F (38% female)	3M, 2F (40% female)
Median age at vaccination	36.5 years (15–48)^a^	—	63 years (60–71)
Median age at infection	—	52 years (7–72)	67 years (64–72)
Median time between vaccination and infection	—	—	3 years (0.5–4)
Median age at sample draw (first sample)	38 years (24–55)	52 years (7–72)	69 years (64–73)
Median time between vaccination and sample draw (first sample)	23 months (3–204)	—	48 months (12–72)
Median time between infection and sample draw (first sample)	—	6 months (1.5–120)	12 months (6–24)
Full TBEV vaccination course?	Yes: 15, No: 0^a^	—	Yes: 5
TBEV vaccine boosters?	Yes: 3, No: 12^a^	—	Unknown: 5
Previous YFV vaccination?	Yes: 11, No: 4^a^	—	—
Previous JEV vaccination?	Yes: 4, No: 11^a^	—	—

^a^ Information not available for four donors

**Fig. 3:**
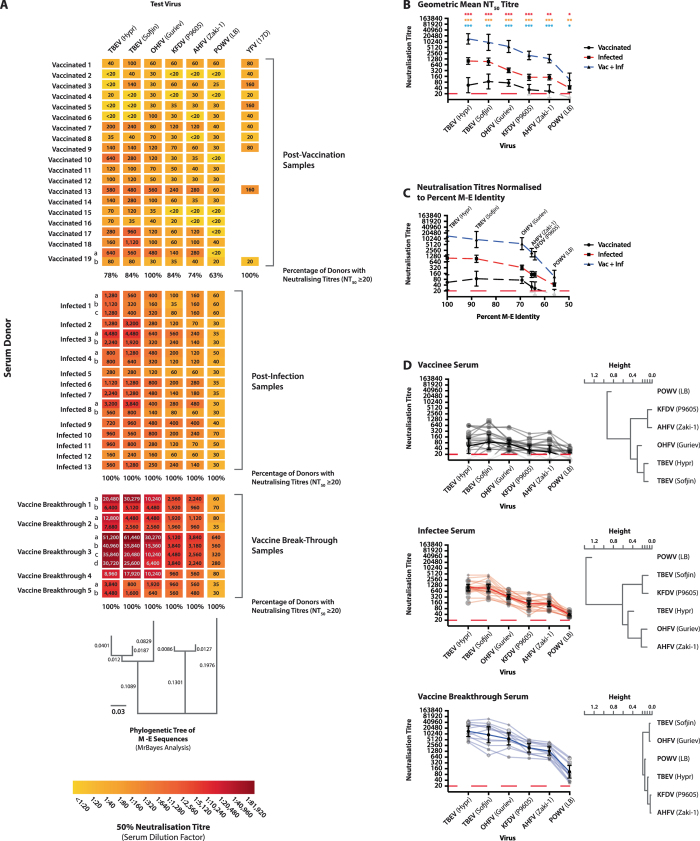
Cross-neutralisation of tick-borne flaviviruses with TBEV vaccinee, infectee and vaccine breakthrough human sera. Six tick-borne flaviviruses (listed in Table 1) were neutralised using human serum samples from TBEV vaccinees, infectees and vaccine breakthroughs, with the NT_50_ neutralisation titres presented in a heatmap (Fig. 3a). Letters a, b, c and d next to donor numbers indicate multiple samples taken at different timepoints. For vaccinees who had also received the YFV vaccine, anti-YFV 17D PRNT_50_ titres were also determined. Percentage of donors with neutralising titres are stated for each exposure type. Test viruses were grouped based upon MrBayes phylogenetic analysis of the M-E amino-acid sequences. The geometric mean neutralisation titre (GMT) and standard error for each virus and exposure type were calculated and plotted both equally spaced (Fig. 3b) and arranged based upon the percent M-E identity with TBEV Hypr (Fig. 3c). Each individual neutralisation profile (Fig. 3d; *faint lines*) and GMT (*bold lines*) were plotted for each exposure type. Test viruses were hierarchically clustered based upon cross-neutralisation by the donor sera. Since the data for some groups violated the equal variance assumption, comparison of neutralising titres between groups was performed using one-way Welch’s analysis of variance with Games-Howell post hoc analysis:*Denotes *p* < 0.05, **Denotes *p* < 0.01 and ***Denotes *p* < 0.001. *Red stars* represent comparison between the vaccinee and infectee donors, *yellow stars* represent comparison between the vaccinee and vaccine-breakthrough donors, while *blue* represents comparison of infectee and vaccine-breakthrough donors

Each of the serum samples was tested against six tick-borne flaviviruses in the microneutralisation tests (Fig. 3a). As can be clearly seen by the heatmaps of the neutralisation titres, substantial cross-neutralisation occurred with each exposure type, although the magnitude of the immune response varied depending on whether the donor was vaccinated or infected: infection generally led to higher neutralising titres than vaccination. When compared to the phylogenetic tree of the M-E sequences from the test viruses, the neutralisation titres decrease with increasing genetic diversity from the European TBEV Hypr.

When the results are analysed and compared based upon exposure type, with geometric mean titre (GMT) and standard error overlaid on the individual data points, the differences in magnitude of the antibody responses between exposure categories become more apparent (Fig. 3b), with statistically significant differences observed between each of the exposure groups for each virus. The three exposure categories show a similar trend towards decreasing neutralisation titres as genetic diversity increases from TBEV Hypr. This is further exemplified when the data are analysed based upon percentage variation in amino-acid identity between M and E of the test virus compared to TBEV Hypr (Fig. 3c): the titres are not substantially lower between the two TBEV strains and OHFV, but fall rapidly past 69% amino-acid identity. There is clearly vaccine-induced neutralisation of KFDV and AHFV, with 84 and 74% of vaccinees showing detectable neutralisation, respectively. POWV is poorly neutralised by the test sera regardless of the exposure category, but especially following vaccination, with only 63% of vaccinees displaying detectable neutralisation titres, and no NT_50_ titres exceeding 1:60. It is clear that there is substantial variability in the individual donor responses to vaccination with regards to cross-neutralisation profiles (Fig. 3d), with the profiles displaying a less-linear trend than those observed with the other exposure types. By contrast, the variation between donors following vaccine breakthrough and, to a lesser extent infection, is substantially lower. Hierarchical clustering of the virus-specific neutralisation profiles, grouping the viruses based upon the overall neutralisation response against them, confirms the variability seen by plotting the individual values: the greater variability in neutralisation profiles seen in the vaccinee and infectee samples corresponds to a greater distance between the viruses in the clustering, while the almost parallel lines for the vaccine breakthrough donors equates to a close relationship by clustering.

**Fig. 4:**
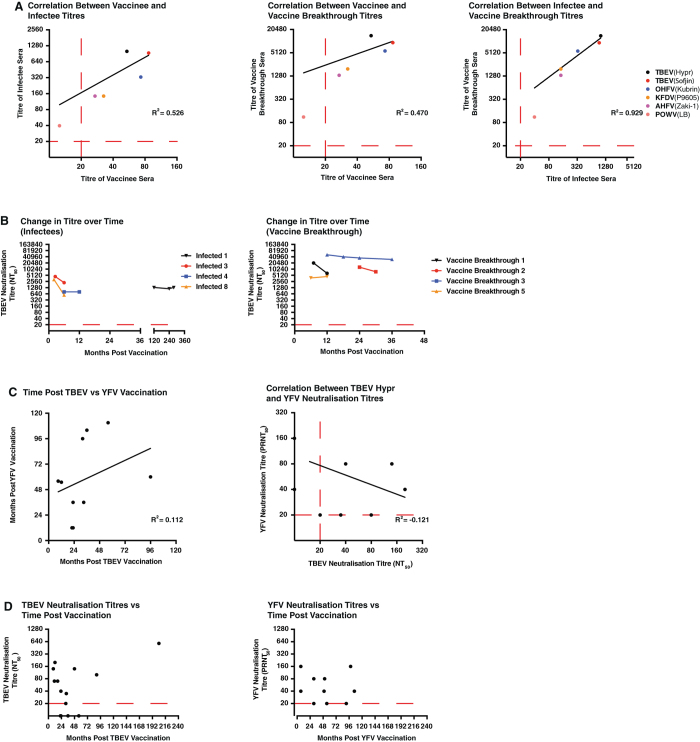
Correlation between exposure types, effect of time on neutralisation titre, and comparison of TBEV and YFV vaccination titres. The geometric mean neutralisation titres for each virus were correlated based upon exposure type (Fig. 4a), with correlation analysis performed using a Log-Log non-linear curve fit in GraphPad Prism 7 (GraphPad, La Jolla, CA). Infectee and vaccine breakthrough donors who had multiple samples from different timepoints were analysed in a temporal manner (Fig. 4b) to determine whether neutralisation titres waned with time. Comparison of TBEV Hypr and YFV 17 vaccine-to-blood draw interval, as well as NT_50_ and PRNT_50_ values, were performed using a Log-Log non-linear curve fit (Fig. 4c). In addition, comparison of TBEV Hypr NT_50_ and YFV 17D PRNT_50_ neutralisation titres with the vaccine-to-blood draw interval was performed (Fig. 4d)

When the nature of cross-neutralisation for each exposure type is compared by correlation (Fig. 4a), it is clear that the response following infection and vaccine breakthrough is very similar (*R* ^2^ = 0.929), while the response following vaccination is more distinct (*R* ^2^ = 0.526 compared to infectees, *R* ^2^ = 0.470 compared to vaccine breakthroughs). This appears to be primarily due to the lower-than-expected neutralisation of TBEV Hypr, a virus often neutralised less efficiently than TBEV Sofjin, and occasionally even less than OHFV. Indeed, if the TBEV Hypr values are removed from the correlation analysis, the correlation coefficients increase substantially (*R* ^2^ = 0.919 compared to infectees, *R* ^2^ = 0.979 compared to the vaccine breakthroughs; data not shown).

For four each of the infectee and vaccine breakthrough donors, samples from multiple timepoints were available for testing to investigate the change in neutralisation titres over time (Fig. 4b). These results failed to reveal any useful results, as there was significant inter-donor variability with no overall trends being apparent in the data.

In order to determine whether apparently poor responders to TBEV vaccination were generally poor at responding to flavivirus vaccination, PRNT_50_ assays were run with yellow fever virus (YFV) 17D for vaccinees who had also received the commercially available YFV vaccine (*n* = 10). Although the intervals between TBEV vaccination and blood draw and YFV vaccination and blood draw were not consistent among the vaccinees (Fig. 4c; *R* ^2^ = 0.122), when TBEV and YFV titres were plotted together (Fig. 4c), there was no clear correlation between anti-TBEV Hypr NT_50_ titre and YFV PRNT_50_ titre (*R* ^2^ = −0.121). Nor was there any significant correlation between the neutralisation titre and the interval between vaccination and blood draw for either TBEV Hypr or YFV 17D (Fig. 4d). These data suggest that those with a poor response to TBE or YFV vaccination do not appear to have an underlying condition limiting their responsiveness to flavivirus vaccines.

## Discussion

The TBE complex is a group of significant human pathogens that share a relatively high degree of genetic similarity. Due to the frequently high virulence associated with these viruses, they have been major targets for vaccine development. However, to date, only TBEV has a licenced vaccine with widespread availability. Although the correlates of protection for flaviviruses have not been thoroughly determined, the current understanding is that neutralising antibodies play an important role. The aim of this study was twofold: firstly, to determine the potential for using the currently available vaccines to protect against other tick-borne flaviviruses for which vaccines are not available; and, secondly, to compare the cross-neutralisation responses following vaccination with those following natural infection or vaccine breakthrough.

Consistent with other reports, the data clearly show that there is significant cross-neutralisation between viruses of the TBE complex.^8–13^ With the MIAF samples, we have added AHFV to the panel of virus/antisera tested in cross neutralisation assays, and the results agree with the virus’ position in the phylogenetic tree as determined by genetic analysis.

Compared to the other flaviviruses tested, OHFV was readily neutralised by each of the MIAFs tested. Moreover, with some of the human vaccinee samples, OHFV was more readily neutralised than even the TBEV strains. This observation has been made in previous studies with human vaccinees, with TBEV vaccination inducing higher (although not statistically significant) neutralisation titres against OHFV than TBEV.^42^ Whether this is indicative of OHFV possessing more broadly reactive neutralising epitopes than other tick-borne flaviviruses, or whether OHFV may be more-readily neutralised by antibodies with lower affinity/avidity, is unclear, but is worth considering with regards to cross-neutralisation following vaccination.

For the most part, the strength of cross-neutralisation responses against different viruses with the MIAFs followed the level of genetic similarity between M and E amino-acid sequences among the viruses (Fig. 2). When the hierarchical clustering of the cross-neutralisation profiles for the different MIAFs is compared to the phylogenetic tree of the M-E sequences, the grouping is similar, with the two TBEV strains grouping with OHFV, and the KFDV and AHFV strains being more distinct. Interestingly, despite 93.2% amino-acid identity with TBEV Hypr, the anti-LIV MIAF did not efficiently neutralise any of the tested viruses other than OHFV.

With the human serum samples, the cross-neutralisation profiles following vaccination, infection or vaccine breakthrough showed similar trends: a gentle decline in titre down to 70% M-E amino-acid identity, then a more rapid decrease in titre below 70% identity (Fig. 3). Accordingly, effective neutralisation following vaccination was observed for the TBEV strains and OHFV; and, to a lesser extent KFDV and AHFV; but not POWV. Indeed, anti-POWV neutralisation titres were relatively lower even in vaccine breakthrough samples that had anti-TBEV titres in excess of 1:1,600, and some as high as 1:61,440. This more rapid decline in neutralisation titre below 70% amino-acid identity is likely due to regions of sequence diversity in domains I and II of the envelope glycoprotein (EI and EII). Studies by Jarmer *et al.* ^45^ have demonstrated that antibody responses (both general and neutralising) following TBEV vaccination and infection are primarily targeted towards this region of the protein and, when the amino-acid sequences of these domains are compared for the viruses, clear regions of dissimilitude are visible, particularly around residues 66–68 and 228–240 (Fig. 1).

The statistically significant differences in magnitude of the titres following the three exposure types likely result from the relative abundance and context of antigen presentation encountered by the immune system. Since the vaccines are inactivated preparations of virus, the amount of antigen available to the immune system is fixed and responses are limited to MHC class II/CD4^+^ T-cell by virtue of the exogenous nature of the antigens.^49^ By contrast, infection allows for a larger and more persistent supply of antigen for as long as there is replicating virus. Active infection of host cells also allows for the presentation of antigens by both MHC class I and class II molecules, thus driving both CD4^+^ and CD8^+^ T-cell responses.^49, 50^ This diversity of immune response following infection compared to vaccination (particularly inactivated vaccines) likely explains the substantially higher neutralisation titres associated with infection.

Vaccine breakthrough appears to be primarily associated with individuals for whom primary vaccination occurred in older age (>60 years). Immune responses in older age have been shown to be less efficient than in younger people, with previous studies demonstrating that while antigen-specific memory B-cells are generated following TBEV vaccination, in older people the numbers of these cells are reduced.^51^ Moreover, the same study suggested that reduced antibody levels in these donors are also associated with an impairment of CD4^+^ helper cell responses.^51^ The combined effect of these impairments may explain why the vaccine breakthroughs occurred. Moreover, vaccine breakthrough donors generally had higher neutralising antibody titres than donors from either of the other exposure groups, with this group displaying an anamnestic response as previously suggested: these donors had a primary antibody response following vaccination, but the response was inefficient and antibody titres were either low, or fell rapidly.^29–31^ Upon exposure to the virus, the comparatively low complement of memory B-cells were sufficiently delayed in recall to allow for infection to occur with resulting clinical disease, but the prior priming of the immune system meant that a cumulative vaccine and post-infection antibody response resulted from the infection.

The results of the human vaccinee serum samples suggest that TBEV vaccination may be appropriate for providing protection against other tick-borne flaviviruses in high-risk individuals, but that the responses to vaccination are highly variable. This variability was not solely related to the post-vaccination interval before sample collection, as evidenced by the poor correlation between titre and post-vaccination interval. Accordingly, it may be appropriate to test vaccinee antibody titres prior to commencement of high-risk activities. Similarly, regular assessment of residual titres should be considered for those deemed to be at high risk of infection to determine whether booster vaccination may be appropriate owing to the apparent variability of post-vaccination dynamics. Neutralisation titres following TBEV vaccination did not correlate with those for YFV vaccination, suggesting that the variability is not due to inherently poor reactivity to flavivirus vaccines, although this comparison is also likely influenced by inherent differences in immunogenicity of inactivated (TBE) vs. live, attenuated (YF) vaccines.

While vaccinee neutralisation titres against KFDV and AHFV were lower than for the TBEV strains and OHFV, 84% of donors demonstrated neutralisation of KFDV and 74% of donors neutralised AHFV, with GMTs of 1:32.5 and 1:27, respectively. By contrast, vaccination with the Indian formalin-inactivated tissue culture-derived KFDV vaccine has been reported to lead to the development of neutralising antibodies in only 57.3% of vaccinees.^37, 38^ This suggests that, unlike the old Soviet formalin-inactivated mouse brain-derived TBEV vaccine which showed little reactivity against KFDV,^41^ the current commercially available TBEV vaccines may be more effective for the prevention of KFDV than even the current KFDV vaccine.

With the recent resurgence of POWV in North America,^52^ and the likely further increase in cases over the coming years associated with increasing temperatures, preventive vaccination against this tick-borne pathogen will likely become more important. However, these studies demonstrate that despite possessing a high degree of amino-acid identity with TBEV Hypr, POWV was not effectively neutralised by the TBEV vaccinee sera utilised, with detectable neutralisation titres (≥1:20) in <70% of donors, and none of the donor possessing neutralisation titres >1:60. As a consequence, vaccination with the commercially-available TBEV vaccines does not appear to be an appropriate approach for the prevention of POWV infection.

In summary, these data demonstrate that there is significant cross-neutralisation of viruses of the TBE complex following TBEV vaccination and/or infection. While the magnitude of the response varied depending on the type of exposure involved, the overall trends were similar. Unlike the formalin-fixed mouse brain-derived TBEV vaccines of old, the current vaccines appear to generate neutralising responses against viruses including KFDV and AHFV, and could, therefore, be considered for the protection of high-risk individuals such as laboratory workers working with these agents on a regular basis. These findings give us a better understanding of the relationship between cross-neutralisation and genetic relationship of tick-borne flaviviruses, with some of these concepts potentially applicable to other related flaviviruses.

## Methods

### Cells and viruses

Virus titration and microneutralisation assays (see below) were performed using mycoplasma-free BHK-SA cells,^53^ owing to their high susceptibility to all of the viruses tested. Viruses were acquired from the WRCEVA at the UTMB. Virus strains used for the study can be found in Table 1, with the published GenBank accession numbers used for sequence comparison. All work with infectious viruses was performed within the Galveston National Laboratory or Robert E. Shope BSL-4 laboratories. Prior to use, each virus stock was titrated using a TCID_50_ approach. Briefly, a 96-well plate was seeded with BHK-SA cells at 3×10^3^ cells/well (a 1:10 dilution) in Dulbecco’s modified Eagle medium (DMEM; Gibco/Thermo Fisher, Waltham, MA) containing 1% Foetal Calf Serum (FCS; HyClone, Logan, UT), 1× Penicillin/Streptomycin (Corning, Manassas, VA), 1× non-essential amino acids (Corning, Manassas, VA), and 1× L-Glutamine (Thermo Fisher, Waltham, MA). Once the cells reached approximately 40% confluence, they were transferred to the BSL-4 facilities at UTMB. Serial tenfold dilutions of virus were prepared in a 96-well plate starting at 10^0^, with triplicate 4-column groups for the calculation of a TCID_50_ value. The plated BHK-SA cells were washed with 1× Dulbecco's phosphate-buffered saline (DPBS; Corning, Manassas, VA) and 100 μl of diluted virus was transferred from each well of the dilution plate to the corresponding well of the cell plate. The plates were incubated at 37 °C with 5% CO_2_ for three days (for TBEV Hypr, TBEV Sofjin, AHFV Zaki-1, AHFV 200300001, and KFDV P9605) or four days (for OHFV Guriev and POWV LB) to allow for the development of cytopathic effect (CPE) within infected wells. After the incubation, the media was removed from the wells, and the cells were stained with 0.25% crystal violet (Sigma-Aldrich, St. Louis, MO) in 10% buffered formalin (Fisher Scientific, Waltham, MA). The plates were fixed for 30 min at 37 °C before the crystal violet was removed and the plates were rinsed with water. TCID_50_ values were calculated using the technique of Reed and Muench.^54^

### Anti-flavivirus MIAFs

MIAFs raised against a range of tick-borne flaviviruses were acquired from the WRCEVA at UTMB. Briefly, the MIAFs were generated mice inoculated three times with material derived from the virus of interest. The first vaccination consisted of irradiated mouse brain preparation in Freund’s Complete adjuvant, the second consisted of irradiated mouse brain preparation in Freund’s Incomplete adjuvant, and the third consisted of live virus in a mouse brain preparation. The MIAFs used in the study were raised against the same strains of virus employed for the neutralisation assays with the exception of OHFV: for the MIAF generation the Kubrin strain was used, while the neutralisation assays were run with the Guriev strain.

### Human serum samples

Human serum samples were collected from donors following vaccination or infection with TBEV. Four serum samples from donors who had received YFV vaccination, but not TBEV vaccination, were used as negative control sera to determine limits of detection (LODs), and to minimise any confounding effect of YFV vaccination on the cross-neutralisation data. Nine serum samples were collected from volunteers who had received a full course of TBEV and YFV vaccinations at UTMB for occupational health purposes. A further nine samples were taken from donors vaccinated with the full course of TBEV vaccine doses at the Paul-Ehrlich-Institut in Germany. TBEV vaccination for all vaccinee donors was performed with either the FSME-Immun or Encepur European vaccines, while YFV vaccination was performed using the live, attenuated YF-Vax vaccine (Sanofi Pasteur, Swiftwater, PA). Original serum samples from one vaccinated donor (two samples), thirteen infected patients (18 samples total), and five vaccine breakthrough patients (11 samples total) were obtained from donors at the University of Ljubljana, Slovenia. Further information about the serum donors can be found in Table 2.

### Ethics statement

The study was assessed by the Institutional Review Board at UTMB, and was deemed “not considered human research” owing to the human serum samples being de-identified prior to delivery for testing and evaluation. Informed consent was obtained from all donors prior to testing.

### Microneutralisation assays

Microneutralisation assays were performed in BSL-4 containment at UTMB using a TCID_50_-based approach similar to that described previously.^43, 55^ In brief, BHK-SA cells were plated in 96-well tissue culture plates as described above. Once the cells reached a confluence of approximately 40%, the plates were transferred to the BSL-4 laboratory for infection. Heat-inactivated (56 °C for 30 min) human serum or MIAF samples were diluted in serial twofold dilutions in a 96-well plate, with 60 μl per well, from a starting dilution of 1:5 in DMEM/1% FCS. Each sample was diluted in quadruplicate to allow for the determination of an NT_50_ value. Titrated virus stocks were diluted in DMEM/1% FCS to a final concentration equivalent to 100 TCID_50_s per 50 μl. Sixty microlitres of diluted virus was added to each well of the serum/MIAF plate to give a serum/MIAF dilution range of 1:10 onward. Back titrations were run each time for each virus. The serum-virus-containing plates were incubated at 37 °C for 30 min to allow for antibody adsorption to virus particles. During incubation, the media was removed from the cell-containing plates, and each well was washed with 1× DPBS (Corning, Manassas, VA). Following the virus-serum/MIAF incubation, the samples were transferred to the cell-containing plate (100 μl per well). The plates were incubated at 37 °C with 5% CO_2_ for three days (for TBEV Hypr, TBEV Sofjin, AHFV Zaki-1, AHFV 200300001 and KFDV P9605) or four days (for OHFV Guriev and POWV LB) to allow for the development of CPE within infected wells. After incubation, the media was removed from the wells, and the cells were stained with 0.25% crystal violet (Sigma-Aldrich, St Louis, MO) in 10% buffered formalin (Fisher Scientific, Waltham, MA). The plates were fixed for 30 min at 37 °C before the crystal violet was removed and the plates were rinsed with water. NT_50_ values were calculated using the technique of Reed and Muench.^54^

### Plaque-reduction neutralisation test (PRNT)

For the samples from vaccinated individuals, neutralisation titres were determined against the YFV 17D vaccine strain using the PRNT approach with a 50% neutralisation endpoint. In brief, 24-well tissue culture plates were plated with Vero cells (ATCC CCL-81) at a concentration of 7×10^4^ cells/well in minimal essential medium (MEM; Gibco/Thermo Fisher, Waltham, MA) containing 10% FCS (HyClone, Logan, UT), 1× Penicillin/Streptomycin (Corning, Manassas, VA), 1× non-essential amino acids (Corning, Manassas, VA), and 1× L-Glutamine (Thermo Fisher, Waltham, MA). Plates were incubated overnight at 37 °C with 5% CO_2_ until the cells were approximately 95% Confluent. Heat-inactivated (56 °C for 30 min) vaccinee serum samples and control anti-YFV 17D mouse immune ascitic (MIAF) samples were diluted 1:10 in MEM containing 2% FCS (12 μl serum with 108 μl medium) in duplicate in the first row of a 96-well plate. Serial twofold dilutions were performed to give a dilution range of 1:10 to 1:640. Sample-free medium was used as a control. To each well of the 96-well plates, 60 μl of medium containing 40 pfu YFV 17D was added (giving a final serum dilution range of 1:20 to 1:1280), followed by thorough mixing by pipetting, and incubation for 60 min at room temperature. During the incubation, the media was removed from the plates with monolayers, and the wells were washed with sterile 1× DPBS (Corning, Manassas, VA). After the incubation was complete, the PBS was removed from the cells, and 100 μl per well of the duplicate, serially diluted samples were added to each of the wells. The serum/virus/cell plates were incubated at room temperature for 60 min with occasional rocking to allow for infection of cells by un-neutralised virus. At the end of the incubation, 1 ml MEM/2% agar overlay was applied to each well, and the plates were incubated for 96 h at 37 °C in an atmosphere of 5% CO_2_. Following the 96 h incubation, the plates were fixed, permeabilised, and immunostained as described elsewhere.^56^ Fifty percent neutralisation titres were determined as the highest dilution of serum that resulted in <50% of the number of foci compared to the average of the control wells. For each of the duplicate values for each serum sample, the highest 50% neutralisation value was reported as long as the duplicate values were within twofold of one another.

### Phylogenetic analysis/heatmap analysis

Amino-acid alignments were performed on sequences from flavivirus mature membrane (M) and envelope (E) proteins using a MUSCLE alignment in Geneious R9 (Biomatters, Auckland, New Zealand). Phylogenetic trees were prepared using the MrBayes plugin for Geneious R9 using a Poisson matrix, a 1,100,000 chain length, and a 100,000 burn-in length. Heatmaps were prepared using Mathematica v10 (Wolfram Research, Champaign, IL), and were edited using Adobe Illustrator (Adobe Systems, San Jose, CA).

### Statistical analysis

GMTs were calculated for each virus using Microsoft Excel (Microsoft, Redmond, WA). For values below the LOD (1:20), an arbitrary value of 1:2 (10% of the LOD) was assigned to allow for the calculation of GMT. Hierarchical clustering analysis was performed on the neutralisation data using Mathematica v10 with the RLink plugin. Correlation analysis was performed using a Log-Log non-linear curve fit in GraphPad Prism 7 (GraphPad, La Jolla, CA). Neutralising titres for each exposure groups were log_2_ transformed and tested using Shapiro-Wilk normality testing and Levene’s test for equal variance in Stata 14 (StataCorp, College Station, TX). Due to violation of the equal variance assumption,comparison of neutralising titres between groups was performed using one-way Welch’s analysis of variance with Games-Howell post hoc analysis in IBM SPSS Statistics v24 (IBM, Armonk, NY).
